# *Hebeloma* in the Malay Peninsula: Masquerading within *Psathyrella*

**DOI:** 10.3897/mycokeys.77.57394

**Published:** 2021-01-28

**Authors:** Ursula Eberhardt, Nicole Schütz, Henry J. Beker, Su See Lee, Egon Horak

**Affiliations:** 1 Staatliches Museum für Naturkunde Stuttgart, Rosenstein 1, 70191, Stuttgart, Germany; 2 Rue Père de Deken 19, B-1040, Bruxelles, Belgium; 3 Royal Holloway College, University of London, Egham, UK; 4 Plantentuin Meise, Nieuwelaan 38, B-1860, Meise, Belgium; 5 Forest Health and Conservation Programme, Biodiversity Division, Forest Research Institute, Kepong, Malaysia; 6 Schlossfeld 17, A-6020, Innsbruck, Austria

**Keywords:** *
Anamika
*, Agaricales, Dipterocarpaceae, ectomycorrhiza, Fagaceae, taxonomy, tropical forests

## Abstract

In 1994 Corner published five new species within the genus *Psathyrella*, all having been collected on the Malay Peninsula between 1929 and 1930. Three of these species belong to the genus *Hebeloma* and with their vinaceous colored lamellae and spore print, when fresh, they belong to H. sect. Porphyrospora. Of these three species, only one, *P. flavidifolia*, was validly published and thus we herewith recombine it as *H. flavidifolium*. The other two species, *P. splendens* and *P. verrucispora*, are synonyms of *H. parvisporum* and *H. lactariolens*, respectively. We also describe a new Malayan species, *H. radicans*, which also belongs to H. sect. Porphyrospora. These findings confirm the western Pacific Rim as a diversity hotspot for H. sect. Porphyrospora. The records described within this paper, represent the first recognition that the genus *Hebeloma*, and indeed that members of the ectomycorrhizal Hymenogastraceae, are present on the Malay Peninsula.

## Introduction

Only a small number of *Hebeloma* species have been described from Asia, most recently *H. parvisporum* from Laos (Eberhardt et al. 2020). In the same paper, H. sect. Porphyrospora was proposed to include species originally described in *Anamika*. This decision was based on morphological and molecular data. The most distinctive features of the section are the predominantly dry cap surface and a spore deposit that is vinaceous red when fresh, but changes to brown without any reddish hue within one year in the herbarium. This color of the fresh spores, and as a result of the spore deposit, also normally causes the fresh mature lamellae to exhibit at least tinges of vinaceous red. This spore deposit color, and the subsequent color change when dried, appears to be restricted to this one section within *Hebeloma*.

The geographical distribution of species within H. sect. Porphyrospora is remarkable. The majority of the species occur in the western Pacific Rim region, with the exception of two species, *H. porphyrosporum*, to date only known from Europe, and *H. sarcophyllum*, to date only recorded from eastern North America (Beker et al. 2016; Eberhardt et al. 2020).

During the course of this research, and our efforts to find relevant information about *Hebeloma* recorded from the Malay Peninsula, we came across a paper by E.J.H. Corner (1993), effectively published 1994 (Corner 1994 [“1993”]), where he described five new *Psathyrella* species from the Malay Peninsula. These taxa have ornamented spores, and Corner followed Pegler and Young (1992), who also included a few species with ornamented spores in *Psathyrella*. Furthermore, *P. splendens* has a membranous persisting veil forming a conspicuous annulus, a feature excluding its position in the current circumscription of genus *Psathyrella* (Örstadius et al. 2015). This and two other of the new species, according to Corner (1994 [“1993”]), do not fit well within the genus. On the one hand, their robust stature might suggest they should be placed in *Lacrymaria* Pat. (which Voto (2019) did for all five of the Corner (1994 [“1993”]) taxa), but while the spores of *Lacrymaria* are black or certainly very dark in mass, at least the three collections with ornamented spores have fuscous purple or vinaceous brown spores. Also, *Lacrymaria* spores have a germ pore, not seen in these collections.

It is now clear that these three species belong to the genus *Hebeloma*. Based on the spore color in fresh material, they are members of H. sect. Porphyrospora. Unfortunately, the publication of two of these species is invalid under Art. 40.7 of the International Code (Turland et al. 2018), as the published description does not specify the herbarium in which the types are conserved.

It does appear that two of these three species, *Psathyrella splendens* and *P. verrucispora*, have been described and classified within *Hebeloma* since Corner’s publication, as *H. parvisporum* and *H. lactariolens*, originally published as *Alnicola lactariolens*. The third taxon, *P. flavidifolia* is recombined here as *H. flavidifolium*. Within this paper we cite seven new *Hebeloma* collections from the Malay Peninsula, collected by one of the authors (E. H.) during 2009 and 2010. Three of these collections are referred to *H. lactariolens*, one to *H. parvisporum*, two to *H. flavidifolium* and one to a species here described as new, *H. radicans*. All collections are from mixed tropical lowland forests dominated by *Dipterocarpus*, *Quercus* and *Lithocarpus*.

Corner (1994 [“1993”]) published detailed descriptions and excellent drawings of *P. splendens* and *P. verrucispora*. However, his description of *P. flavidifolia* is rather brief and has very little microscopic detail. He writes: “*P. flavidifolia*, is imperfectly known from one collection and is included in order that it may be rediscovered”. He goes on to say: “I describe this fungus, even though my notes on microscopic details are so imperfect, because it indicates an ally of *P. splendens*. It may be rare because I found it but once and, then, it puzzled me and became *Hebeloma* in my notes”. It appears that Corner already guessed that perhaps this taxon belonged within *Hebeloma*. Based on two new collections from the Malay Peninsula, we can now provide a much more detailed description and photographs of this mushroom. The description of *Hebeloma radicans* is based on a single collection. Although this is unfortunate, we have decided to go ahead with the description of this new species, anticipating that the knowledge of this species will advance its rediscovery and that of related taxa.

## Materials and methods

Basidiomes were collected, dried and accessioned at the fungus herbarium of the Forest Research Institute Malaysia (**FRIM**) with duplicates in the collection of E. Horak at the herbarium of the Eidgenössische Technische Hochschule Zürich (**ZT**). Type material of the Corner species was obtained from the herbarium of the Royal Botanic Garden of Edinburgh (**E**).

Sequence data were obtained from dried specimens by direct sequencing following methods detailed in Eberhardt et al. (2016) and Cripps et al. (2019) for ITS and Vesterholt et al. (2014) for *MCM7* (a DNA replication licensing factor). Sequence data were generated by LGC Genomics (Berlin, Germany). Sequences were edited using Sequencher vs. 4.8 (Gene Codes Corp., Ann Arbor, Michigan). Newly generated sequences were accessioned to GenBank (MT832016–MT832022 and MT832328–MT832331).

*Flammula alnicola* was used for rooting, and two species of *Alnicola* [*Naucoria* fide Species Fungorum (Index Fungorum Partnership 2019) accessed 13 Dec 2019] (*A. amarescens* and *A. salicis*) were used as additional outgroups. Members of the genus *Hebeloma* are represented by material, including type material, used in earlier publications (Beker et al. 2016; Eberhardt et al. 2020) and listed in Table 1. Material of all sequenced collections (apart from MEL 2382694) was available for examination.

**Table 1.** Sequences used in the analysis. Herbarium abbreviations follow Index Herbariorum and are given in capital letters followed by a space or hyphen and the herbarium number. Private collections are indicated by the lack of a space between the letters and numbers. MO refers to https://mushroomobserver.org/

Species	Country	HJB database reference	Voucher	GenBank acc. no. ITS	GenBank acc. no. MCM7
*Alnicola amarescens* (Quél.) R. Heim & Romagn.	Switzerland	HJB11116	HJB11116	MK961996†	MK961952†
*Alnicola salicis* (P.D. Orton) Bon	U.K.	HJB14745	HJB14745	MK962001†	MK961960†
*Flammula alnicola* (Fr.) P. Kumm.	Germany	–	GLM-F045994	MK957190†	MK961971†
*Hebeloma aestivale* Vesterh.	U.K.	HJB9291	HJB9291	KT218221‡	MK961944†
*H. alboerumpens* Vila & al.	Spain	HJB13021	JVG1090114-15	JQ751220§	JQ751104§
*H. alpinum* (J. Favre) Bruchet	Switzerland	HJB11132	HJB11132	KM390590|	KM390046|
*H. aminophilum* R.N. Hilton & O.K. Mill.	New Zealand	HJB10682	PDD 102982 (PL14504)	MK961993†	MK961949†
*H. aminophilum*	Australia	HJB16823	HO 586929	MK962007†	MK961966†
H. aminophilum f. hygrosarx B.J. Rees	Australia	HJB1000297	PERTH 06659152	MK962016†	MK961969†
*H. angustilamellatum* (Zhu L. Yang & Z.W. Ge) B.J. Rees	China	HJB1000408	HKAS 42927	AY575919¶	–
*H. angustilamellatum*	Thailand	HJB12251	GENT RW07-470	MK961997†	MK961953†
*H. angustilamellatum*	Laos	HJB14851	HNL 501000	MK962003†	MK961962†
*H. angustilamellatum*	Laos	HJB17006	HNL 501053	MK962010†	–
*H. bulbiferum* Maire	Croatia	HJB13083	TUR-A 177060	KT218422‡	MK961956†
*H. cavipes* Huijsman	Spain	HJB9433	HJB9433	KT217362#	KT216685#
*H. celatum* Grilli, U. Eberh. & Beker	Germany	HJB13621	BR 5020184119676	KT218446‡	MK961957†
*H. crustuliniforme* (Bull.) Quél.	Spain	HJB11237	HJB11237	JN943870††	KF309440|
*H. cylindrosporum* Romagn.	Spain	HJB11427	C-F-44748	FJ769365‡‡	MT832328
*H. cylindrosporum*	France	HJB12763	HJB12763	JQ751210§	JQ751106§
*H. dunense* L. Corb. & R. Heim	Belgium	HJB14141	AdH11031	KY271835§§	MK961959†
*H. flavidifolium*	Malaysia	HJB13504	E. Horak 13404 (ZT)	MT832021	–
*H. flavidifolium*	Malaysia	HJB13505	E. Horak 13406 (ZT)	MT832022	–
*H. ifeleletorum* Kropp	American Samoa	HJB1000386	UTC 00235643	MK962019†	MK961970†
*H. indicum* (K.A. Thomas & al.) B.J. Rees	India	HJB1000384	IB 19971307	AF407163||	–
*H. indicum*	India	HJB12902	IB 19991200	MK961999†	MK961955†
*H. khogianum* Bresinsky	New Caledonia	HJB1000388	M-0124631	GU591635¶¶	–
*H. lactariolens* Clémençon & Hongo) B.J. Rees & Orlovich	Japan	–	LAU HC88/95	AY818352¶	–
*H. lactariolens*	China	–	HMAS 280191	KX513590†††	–
*H. lactariolens*	Malaysia	HJB13363	E. Horak 12796 (ZT)	MT832017	MT832330
*H. lactariolens*	Malaysia	HJB13365	E. Horak 13287 (ZT)	MT832019	–
*H. lactariolens*	Malaysia	HJB13503	E. Horak 13381 (ZT)	MT832020	MT832331
*H. laterinum* (Batsch) Vesterh.	France	HJB13703	HJB13703	MK962000†	MK961958†
*H. mediorufum* Soop	New Zealand	HJB10689	PDD 102983 (PL51404)	KM390552|	KM390037|
*H. mediorufum*	New Zealand	HJB10688	PDD102995 (PL167404)	KM390572|	KM390042|
*H. mesophaeum* (Pers.) Quél.	Iceland	HJB11050	HJB11050	MK961995†	MK961951†
*H. parvisporum* Sparre Pedersen & al.	Laos	HJB14850	HNL 501009	MK962002†	MK961961†
*H. parvisporum*	Laos	HJB14852	HNL 500968	MK962004†	MK961963†
*H. parvisporum*	Laos	HJB17004	HNL 500914	MK962008†	–
*H. parvisporum*	Laos	HJB17005	HNL 500984	MK962009†	–
*H. parvisporum*	Laos	HJB17007	HNL 500884	MK962011†	–
*H. parvisporum*	Malaysia	HJB13362	E. Horak 12795 (ZT)	MT832016	–
*H. plesiocistum* Beker & al.	Spain	HJB11514	JVG1021214-5	EU570170‡‡‡	JQ751115§
*H. porphyrosporum* Maire	Italy	HJB10344	HJB10344	MK961992†	MK961947†
*H. porphyrosporum*	Spain	HJB10767	HJB10767	MK961994†	MK961950†
*H. radicans*	Malaysia	HJB13364	E. Horak 13265 (ZT)	MT832018	–
*H. radicosum* (Bull.) Ricken	Belgium	HJB10262	HJB10262	MK961990†	MK961945†
*H. radicosum*	Italy	HJB10314	HJB10314	MK961991†	MK961946†
*H. sarcophyllum* (Peck) Sacc.	U.S.A.	HJB15696	DPL 10569	MK962005†	MK961964†
*H. sarcophyllum*	U.S.A.	HJB17783	MO301904	MK962014†	–
*H. sinapizans* (Paulet) Gillet	U.K.	HJB10628	HJB10628	JQ751191§	JQ751119§
*H. sinapizans*	U.K.	HJB10751	HJB10751	JQ751193§	JQ751121
*H. subvictoriense* B.J. Rees	Australia	HJB1000299	MEL 2331640	MK962017†	–
*H. syrjense* (P. Karst.) P. Karst.	France	HJB12064	HJB12064	JQ751206§	JQ751122§
*H. syrjense*	Finland	HJB12396	C 26197F	JQ751218§	JQ751123§
*H. theobrominum* Quadr.	Estonia	HJB10009	HJB10009	EU570181‡‡‡	JQ751124
*H. theobrominum*	Belgium	HJB10063	HJB10063	FJ816623§§§	JQ751125§
*H. vaccinum* Romagn.	Belgium	HJB9965	HJB9965	KT217371#	KT216689#
*H. velutipes* Bruchet	France	HJB10547	HJB10547	EU570174‡‡‡	MK961948†
*H. velutipes*	U.K.	HJB10483	HJB10483	EU570175‡‡‡	MT832329
*H. vesterholtii* Beker & U. Eberh.	Italy	HJB10339	HJB10339	FJ816629, FJ816630§§§	JQ751132
*H. vesterholtii*	Italy	HJB11869	HJB11869	FJ943239, FJ943240§§§	JQ751135§
*H. victoriense* A.A. Holland & Pegler	New Zealand	HJB12401	PDD 93802 (PL3408)	MK961998†	MK961954†
*H. victoriense*	Australia	HJB16704	HO 586713	MK962006†	MK961965†
*H. vinosophyllum* Hongo	Japan	HJB17411	MO287712 (UK323)	MK962012†	MK961967†
*H. vinosophyllum*	Japan	HJB17413	MO299315 (UK347)	MK962013†	MK961968†
*H. westraliense* Bougher & al.	Australia	HJB1000134	PERTH 01012665	MK962015†	–
*H. youngii* B.J. Rees	Australia	–	MEL 2382694	KP012873|||	–
*H. youngii*	Australia	HJB1000343	BRI AQ669300	MK962018†	–

† Eberhardt et al. (2020); ‡ Grilli et al. (2016); § Eberhardt et al. (2013); | Eberhardt et al. (2015); ¶ Yang et al. (2005); # Eberhardt et al. (2016); †† Schoch et al. (2012); ‡‡ Vesterholt et al. (2009); §§ Beker et al. (2018); || Thomas et al. (2002); ¶¶ Rees et al. (2013); ††† Wei et al. 06 Jul 2016, no reference found; ‡‡‡ Eberhardt et al. (2009); §§§ Eberhardt and Beker (2010); ||| Bonito et al. 19 Oct 2014, no reference found.

Sequence alignments were done online in mafft using the E-INS-i option (Katoh et al. 2017) for ITS and ‘auto’ for *MCM7 data*. Alignments were viewed and reformatted using aliview 1.24 (Larsson 2014). Maximum likelihood (ML) analyses of single locus alignments were calculated in raxml 8.2.10 (Stamatakis 2014) using the raxml-Gui interface 2.0 (Silvestro and Michalak 2012; Edler et al. 2019), with the GTRGAMMA option, 10 searches for the best ML tree, using the MRE option to limit the number of rapid bootstrap replicates.

The compatibility of the two loci was accessed following the principle of Kauff and Lutzoni (2002), assuming a conflict to be significant if two different relationships for the same set of taxa, one being monophyletic and the other non-monophyletic, are supported by bootstrap with more than 75% in ML analyses.

The datasets were then concatenated and subdivided into five partitions, ITS and four *MCM7* partitions, the exon in three partitions by codon position and the intron. In IQ tree 2.0.6, the best partitioning scheme and the best likelihood models were determined under the Bayesian information criterion (Lanfear et al. 2012, 2014: Kalyaanamoorthy et al. 2017). This scheme and the selected models were used for ML tree construction (Nguyen et al. 2015; Chernomor et al. 2016). A bootstrap analysis was run in 500 replicates.

A Bayesian inference (BI) analysis was run with mrbayes 3.2.6 (Ronquist et al. 2012) on CIPRES (Miller et al. 2012). The BI analysis was done unpartitioned in two runs with four chains including one heated chain each using the GTRINVGAMMA model and a uniform prior and sampling one tree of each run every 10,000 generations. The analysis was stopped automatically after 4.28 mio generations. The first 25% of trees were discarded as burnin for calculating posterior probabilities.

Trees were visualized using FigTree 1.4.4 (Rambaut 2006–2018) and submitted to TreeBASE (http://purl.org/phylo/treebase/phylows/study/TB2:S26715). Relationships between species are termed “fully supported”, if bootstrap support is 100% or posterior probability is 1, respectively; and “supported” if bootstrap support ≥ 75% and posterior probabilities ≥ 0.95.

Details of morphological analyses were provided in Beker et al. (2016). For each collection at least 50 spores were measured in Melzer’s reagent, excluding the apiculus. The maximum length and width of each spore was measured, and its Q value (ratio of length to width) calculated. Average length, width, and Q value were calculated and recorded alongside the median, standard deviation, and 5% and 95% percentiles. The assessment and coding of spore characters followed Beker et al. (2016) and Vesterholt (2005). The average width of the widest part of the cheilocystidium in the vicinity of the apex appears to be an important character in the separation of species within *Hebeloma* (Vesterholt 2005). It is also important, when determining this average width near the apex, not to be selective with regard to the cystidia chosen for measurement. To determine the average width at the apex, about 100 cheilocystidia were measured on the lamella edge. For other measurements, around 20 cheilocystidia, separated from the lamella edge, were measured from each collection. Because of the complex shapes of the cheilocystidia, four measurements were made: length, width at apex (A), width at narrowest point in central region (M), and maximum width in lower half (B). The measurements were given in this order, and an average value was calculated for each of these measurements. For each cheilocystidium the ratios A/M, A/B, and B/M were calculated and averaged across all cheilocystidia measured. Measurements were made in 5% KOH and Melzer’s reagent. For all other details with regard to our methodology, see Beker et al. (2016). Each collection studied has a database record number associated with that collection; we give these numbers as we intend to make the database publicly available.

## Results

We obtained ITS data for all recent collections from Malaysia and in addition *MCM7* data for Malaysian *H. lactariolens*. No sequence information could be obtained from Corner’s material. The datasets included 68 ITS and 49 *MCM7* sequences (Table 1). Bootstrap support was based on 350 or 300 replicates, respectively. The single locus ML results obtained under the GTRGAMMA model (See TreeBase submission) were fully compatible.

**Figure 1.**
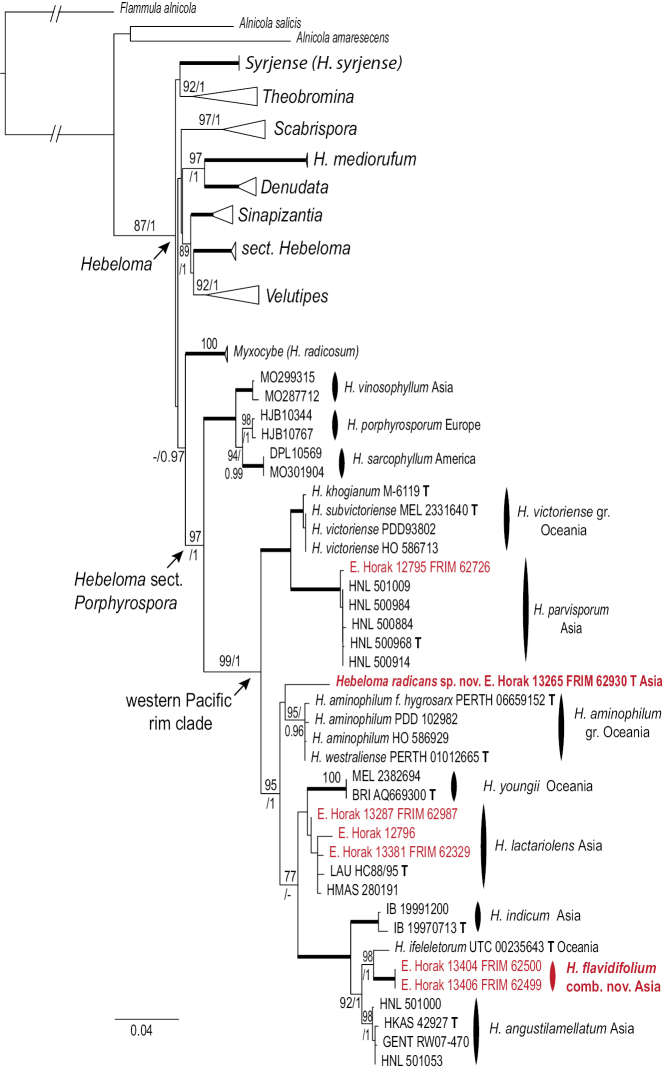
ML topology of concatenated ITS and *MCM7* sequences of *Hebeloma* and *Alnicola*. *Flammula alnicola* is used for rooting purposes. Bootstrap support based on 500 replicates and posterior probabilities based on a BI analysis are indicated at the branches. Assignment of species to sections follows Beker et al. (2016). Sequences in red are from Malaysian collections discussed in this paper. **T** indicates type collections. Thick branches indicate full support. AS – Asia, EU – Europe, NA – North America, O – Oceania, gr. – group.

The concatenated dataset included 1439 sites that were analyzed in three partitions with three different models (ITS: GTR+F+I+G4; MCM7 1^st^ and 3^rd^ position: K3P+I; MCM7 2^nd^ and intron: K2P+I) in the ML tree reconstruction. Bootstrap support was based on 500 replicates. The topology of the ML tree is shown in Fig. 1. The consensus tree resulting from the BI analysis differed from the depicted ML tree only at few supported parts of the tree (see TreeBase submission). Posterior probabilities were based on 642 trees and included in Fig. 1.

**Figure 2.**
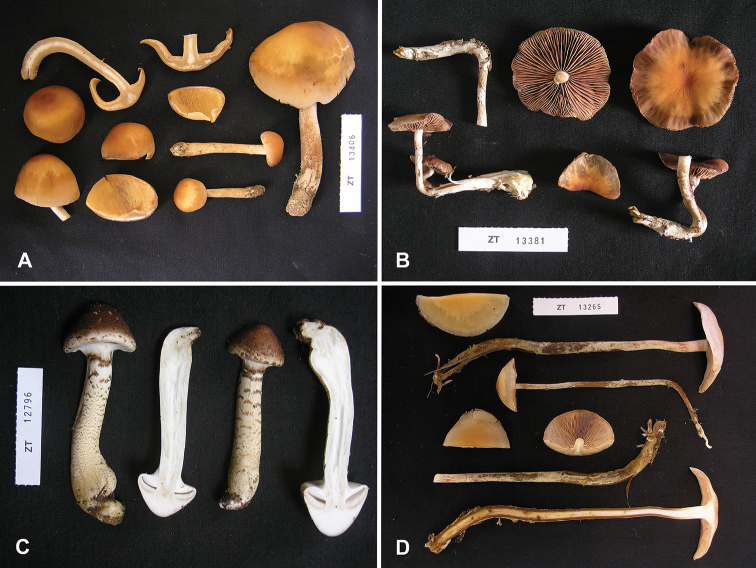
Macroscopic features **A***Hebeloma flavidifolium* (E. Horak 13406) **B***H. lactariolens* (E. Horak 13381) **C***H. parvisporum* (E. Horak 12796) **D***H. radicans* holotype (E. Horak 13265). Photographs E. Horak.

All of the Malaysian collections are included in the clade corresponding to H. sect. Porphyrospora and there within the western Pacific rim clade. The clade of the species *H. flavidifolium* received full bootstrap and posterior probability support as does the clade of *H. parvisporum*. In the ML reconstruction, *Hebeloma lactariolens* is paraphyletic in relation to the sequences of the Oceanic species *H. youngii*, which are monophyletic and receive full support. In the BI result, *H. lactariolens* is monophyletic, but unsupported and in a weakly (0.96 posterior probability) supported sister clade relationship with the clade of *H. angustilamellatum*, *H. flavidifolium*, *H. ifeleleretorum* and the *H. indicum* clade. The Malaysian collections that we refer to as *H. flavidifolium*, *H. lactariolens*, and *H. parvisporum* (Fig. 2) are morphologically and molecularly congruous with each other and other collections from the respective species. The only representative of *H. radicans* is morphologically and molecularly incongruous with all other known species of fungi.

## Taxonomy

We include four species collected from the Malay Peninsula. Three of these have previously been described as *Psathyrella*. Two of these species, *P. splendens* and *P. verrucispora*, were invalidly published but have since been validly published within *Hebeloma*, as *H. parvisporum* and *H. lactariolens*, respectively. The third of these species, *Psathyrella flavidifolia* was validly published and here we recombine it as a *Hebeloma*. Finally, we describe a fourth *Hebeloma* from the Malay Peninsula, *Hebeloma radicans*, as new.

### Hebeloma flavidifolium

Taxon classificationFungiAgaricalesHymenogastraceae

(Corner) Beker & U. Eberh. comb. nov.

81AA558B-2F48-5316-A0E1-A9741CE7BD6C

838406

**Figure 3.**
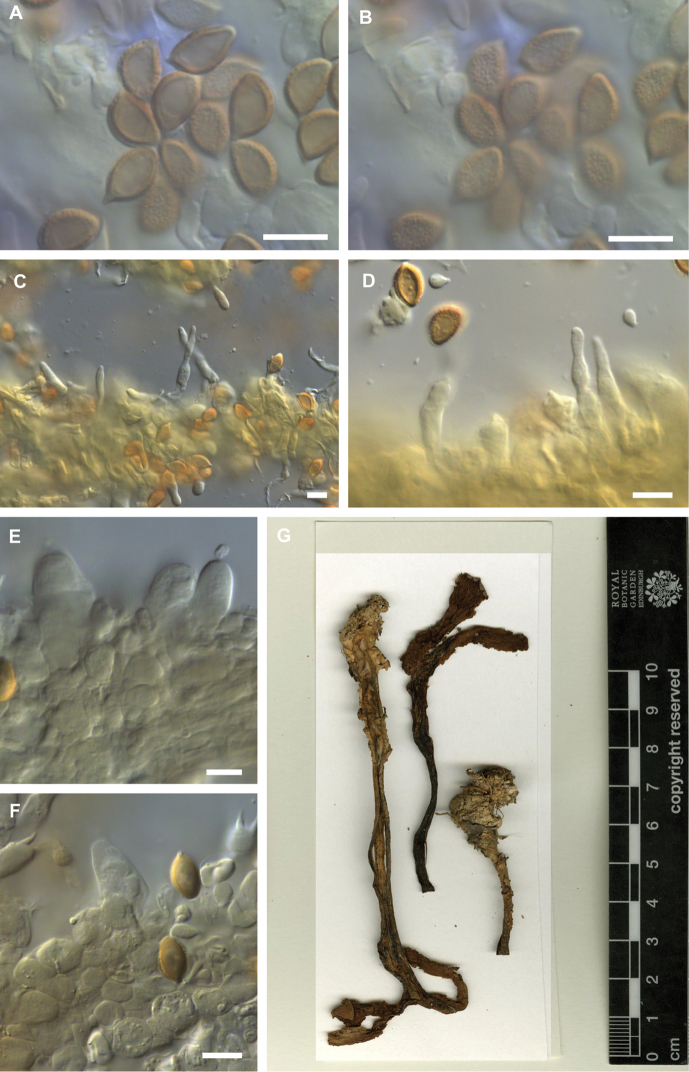
Microscopic features of *Hebeloma flavidifolium* holotype (E 00204812) **A** spores in Melzer’s reagent ×1600 **B** spore ornamentation in Melzer’s reagent ×1600 **C** cheilocystidia in Melzer’s reagent ×500 **D** cheilocystidia in Melzer’s reagent ×1000 **E** cheilocystidia in KOH ×1000 **F** pleurocystidia in KOH ×1000. Scale bars: 10 µm (**A–F**). Photographs H.J. Beker. **G** Exsiccata (a section of photograph from http://data.rbge.org.uk/herb/ E 00204812 provided by the Royal Botanic Garden Edinburgh).

**Figure 4.**
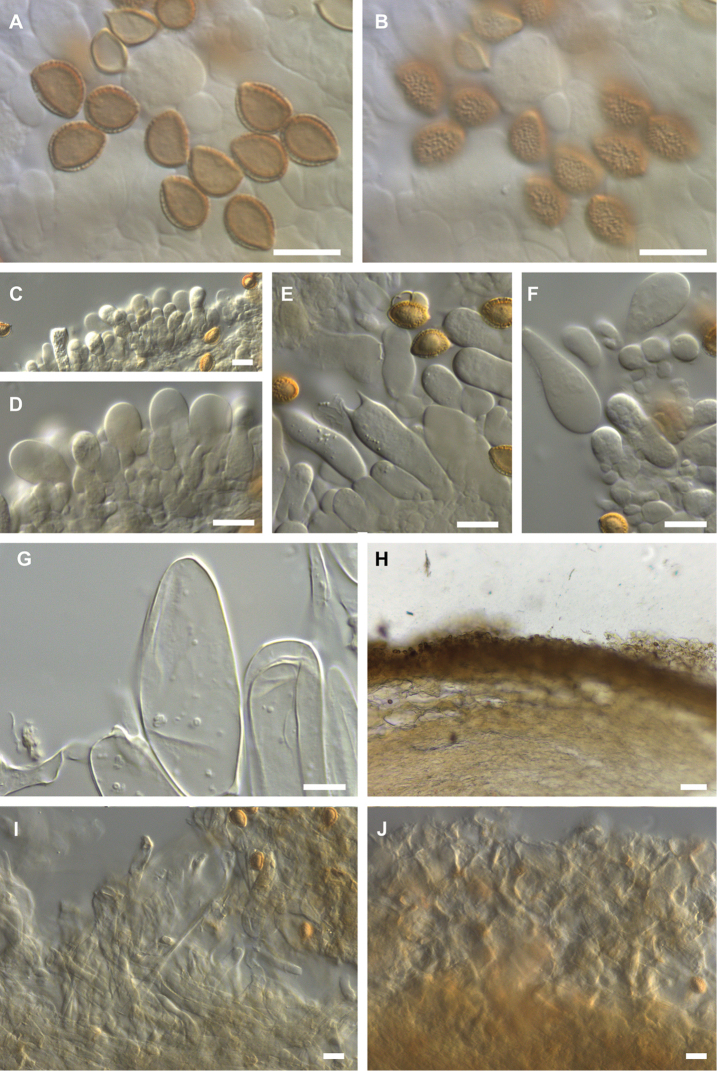
Microscopic features of *Hebeloma flavidifolium* (E. Horak 13406) **A** spores in Melzer’s reagent ×1600 **B** spore ornamentation in Melzer’s reagent ×1600 **C** cheilocystidia in KOH ×500 **D** cheilocystidia in KOH ×1000 **E** basidium in KOH ×1000 **F** pleurocystidia in KOH ×1000 **G** caulocystidium in KOH ×1000 **H** ixocutis section (showing thin gelatinous epicutis) in KOH ×125 **I** epicutis hyphae in KOH ×500 **J** subcutis below epicutis in KOH ×500. Scale bars: 10 µm, 100 µm (**H**). Photographs H.J. Beker.

**Figure 5.**
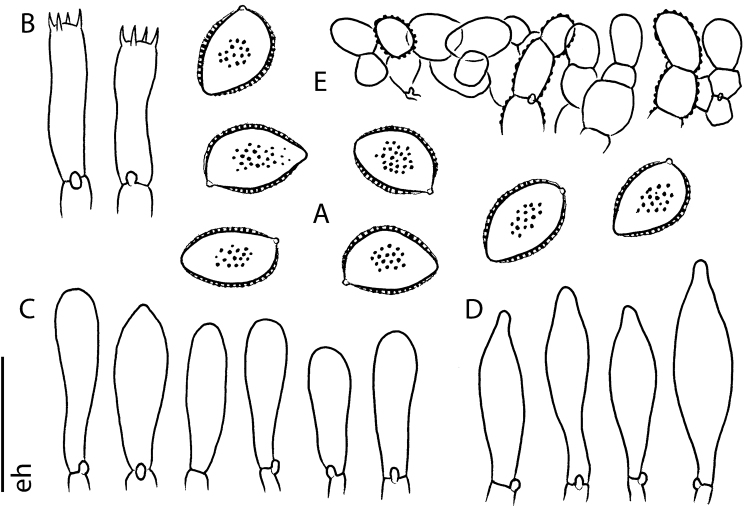
Microscopic features of *Hebeloma flavidifolium* (E. Horak 13406) **A** spores ×2000 **B** basidia ×1000 **C** cheilocystidia ×1000 **D** pleurocystidia ×1000 **E** pileipellis (section of subcutis below epicutis) ×500. Scale bar: 10 µm ×2000, 20 µm ×1000 and 40 µm ×500. Drawing E. Horak.

Figures 2A , 3 , 4 , 5

**Basionym.**Psathyrella flavidifolia Corner, Gdns’ Bull., Singapore 45(2): 339 (1994) [“1993”].

#### Homotypic synonym.

*Lacrymaria flavidifolia* (Corner) Voto, Boll. Assoc. micol. ecol. Romana 107(2): 94 (2019).

#### Type.

Malaysia. Pahang State: Raub district, Bukit Fraser (Fraser’s Hill), ca. 1200 m a.s.l., *Quercus* woodland, 25 Nov 1930, E.J.H. Corner (holotype: E! [E 00204812]; database reference HJB19600).

#### Description.

Basidiomes scattered. Pileus 35–105 mm wide, convex to broadly umbonate; surface dry, sometimes rugulose, occasionally striate at the margin, usually with veil remnants on the margin; cuticle color predominantly cinnamon brown to orange brown (6C5, 7C7) in the center with paler margin, dark beige to tan (5B3); pileus margin strongly involute when young, hygrophanous. Lamellae adnate, often with decurrent tooth, 2–3 mm broad, crowded, thin, with approx. 80–90 full length lamellae and 2–3 lamellules between the lamellae, off-white to cream or yellow-grey when young, later becoming more pinkish or grayish red to purplish and eventually vinaceous to purple-brown or brown following spore maturity; edges weakly fimbriate and white; the white edge remains when the basidiome is dried but the reddish brown color of the lamellae disappears with time. Stipe 50–120 mm long and with central width 5–12 mm, cylindrical sometimes tapering or clavate towards the base, not rooting, occasionally with mycelial cords at the base; white or alutaceous; surface dry, fibrillose, pruinose in the upper part, not discoloring with handling, becoming hollow with age. Flesh whitish, hardly discoloring where bruised. Odor indistinct to raphanoid; taste bitter. Spore vinaceous cinnamon becoming chocolate brown. Exsiccata with no particular characteristics.

Basidiospores based on at least 50 spores from each of three collections, 5% to 95% percentile range 8.9–11.4 × 5.6–7.1 µm, with median 9.7–10.6 × 6.1–6.7 µm and av. 9.6–10.6 × 6.1–6.6 µm with av. S. D. length 0.47 µm and width 0.33 µm; Q value 5% to 95% percentile range 1.43–1.72, with median 1.53–1.58 and av. 1.53–1.59 with av. S. D. 0.07; amygdaloid, occasionally limoniform with small apiculus and rounded apically, with a distinct thinning of the apical wall, without guttules, usually very strongly ornamented, warty, with a strongly and distinctly loosening perispore on almost every mature spore and strongly dextrinoid, becoming medium brown in Melzer’s reagent, sometimes deep brown, ((O3) O4; P3; D3 (D4)); spore color under the light microscope distinctly brown. Basidia av. dimensions 19–33 × 6–9 µm, cylindrical to clavate, without pigmentation, 4-spored. Cheilocystidia irregular, cylindrical to ventricose, often pyriform or napiform often mucronate or rostrate, even lanceolate (as shown in Fig. 3c for example) sometimes septate with width near apex (excluding any rostrum) 5% to 95% percentile range 5.4–10.2 µm, with median 5.6–8.4 µm and av. 5.7–8.6 µm with av. S.D. 0.94; and av. overall measurements 26–29 × 5.7–8.6 × 6.6–9.7 × 5.8–7.5 µm av. Cheilocystidium av. ratios A/M: 0.9–0.91, A/B: 0.77–1.6, B/M: 0.61–1.35. Pleurocystidia present, and abundant, and similar to cheilocystidia, but more often mucronate. Caulocystidia resembling the cheilocystidia but tending to be more cylindrical and longer up to 60 µm. Pileipellis an ixocutis with a very thin epicutis only about 30 µm thick, with gelatinized hyphae, sometimes encrusted, up to 6 µm wide. Subcutis, below the epicutis, orange-brown and the trama below the cutis made up of isodiametric cells up to 17 µm wide. Clamp connections at septa present throughout the basidiome.

#### Distribution.

So far known only from Bukit Fraser (Fraser’s Hill), Malaysia.

#### Ecology.

The recent collections were found scattered in lowland dipterocarp-oak woodland on the side of the path in tropical rain forest with *Quercus*.

#### Additional material examined.

Malaysia. Pahang State: Raub district, Bukit Fraser (Fraser’s Hill), Jalan Girdle, ca. 1000 m a.s.l., 3.71°N, 101.74°E, *Quercus* woodland, 26 Apr. 2010, E. Horak 13406 (collection E. Horak at ZT, FRIM [FRIM 62499]; database reference HJB13505); Pahang State: Raub district, Bukit Fraser (Fraser’s Hill), Jalan Girdle, ca. 1000 m alt., 3.71°N, 101.74°E, *Quercus* woodland, 26 Apr. 2010, E. Horak 13404 (collection E. Horak at ZT, FRIM [FRIM 62500]; database reference HJB13504).

#### Remarks.

Given Corner’s original description almost totally lacked any microscopic information, we present a full description here based on the holotype plus two more recent collections from roughly the same location, both collected by E. Horak. Morphologically, this species most closely resembles *Hebeloma angustilamellatum*, originally described from the Yunnan province of China (Yang et al. 2005) and also recorded from northern Thailand and Laos (Table 1, Fig. 1), from which it can be distinguished morphologically by the very strongly ornamented spores (O4), conspicuous even without immersion (those of *H. angustilamellatum* are O3, so distinctly ornamented but not conspicuous without immersion) and the less conspicuous annulus on the fibrillose stipe of mature basidiomes (*H. angustilamellatum* has a more persistent annulus, always present, and a stipe, with scattered fibrillose scales, consistently present.) Phylogenetically, based on ITS and *MCM7*, *H. flavidifolium* is a sister species of *H. ifeleleretorum* described from Samoa, but all three form a cluster in Fig. 1 that received full posterior probability and 92% bootstrap support.

### Hebeloma lactariolens

Taxon classificationFungiAgaricalesHymenogastraceae

(Clémençon & Hongo) B.J. Rees & Orlovich, Mycologia 105: 1055 (2013).

A5E58920-E487-555D-ACED-55A522F00CB0

Figures 2B , 6

#### Type.

Japan. Shiga-ken: Otsu-shi, Tomikawa, ca. 180 m a.s.l., 34.9001°N, 135.9489°E, *Pinus* sp., *Quercus* sp., 15 Aug 1988, T. Hongo, H. Clémençon HC88/95 (holotype TNS! [TNS-F-237670]; isotype LAU; database reference HJB1000383; ITS GenBank acc. no. AY818352).

#### Homotypic synonyms.

*Alnicola lactariolens* Clémençon & Hongo, Mycoscience 35(1): 25 (1994). *Anamika lactariolens* (Clémençon & Hongo) Matheny, Mycol. Res. 109(11): 1262 (2005).

#### Heterotypic synonyms.

*Psathyrella verrucispora* Corner, Gdns’Bull., Singapore 45(2): 344 (1994) [1993], nom. inval., Art. 40.7 ≡ *Lacrymaria verrucispora* (Corner) Voto, Boll. Assoc. micol. ecol. Romana 107(2): 95 (2019), nom. inval., Art. 40.7. Type: Singapore. Malay Peninsula, Aug. 1929, E.J.H. Corner (holotype E! [E 00204780]; database reference HJB19598).

#### Other material examined.

Malaysia. Johor State: Mersing district, Endau-Rompin Selai, Endau-Rompin (Johor) National Park, Camp Lubuk Tapah, ca. 130 m a.s.l., 2.2976°N, 103.1351°E, with *Dipterocarpus*, 19 Mar. 2009, E. Horak 12796 (collection E. Horak at ZT, FRIM [FRIM 62726]; database reference HJB13363); Johor State: Kluang district, Endau-Rompin Peta, Endau-Rompin (Johor) National Park, trail to Upeh Guling, ca. 40 m a.s.l., 2.5230°N, 103.3611°E, in woodland with *Dipterocarpus* and *Quercus*, 4 Sept. 2009, E. Horak 13287 (collection E. Horak at ZT, FRIM [FRIM 62987]; database reference HJB13365); Negeri Sembilan State: Jelebu district, Simpang Pertang, Pasoh Forest Reserve, ca. 165 m a.s.l., 2.7264°N, 102.0783°E, in woodland, 20 Apr. 2010, E. Horak 13381 (collection E. Horak at ZT, FRIM [FRIM 62329]; database reference HJB13503). SINGAPORE. Malay Peninsula, (E! [E 002048240]; database reference HJB19652), this is just a spore print collected by E.J.H. Corner that may be from the intended type of *Psathyrella verrucispora*.

#### Remarks.

**Figure 6.**
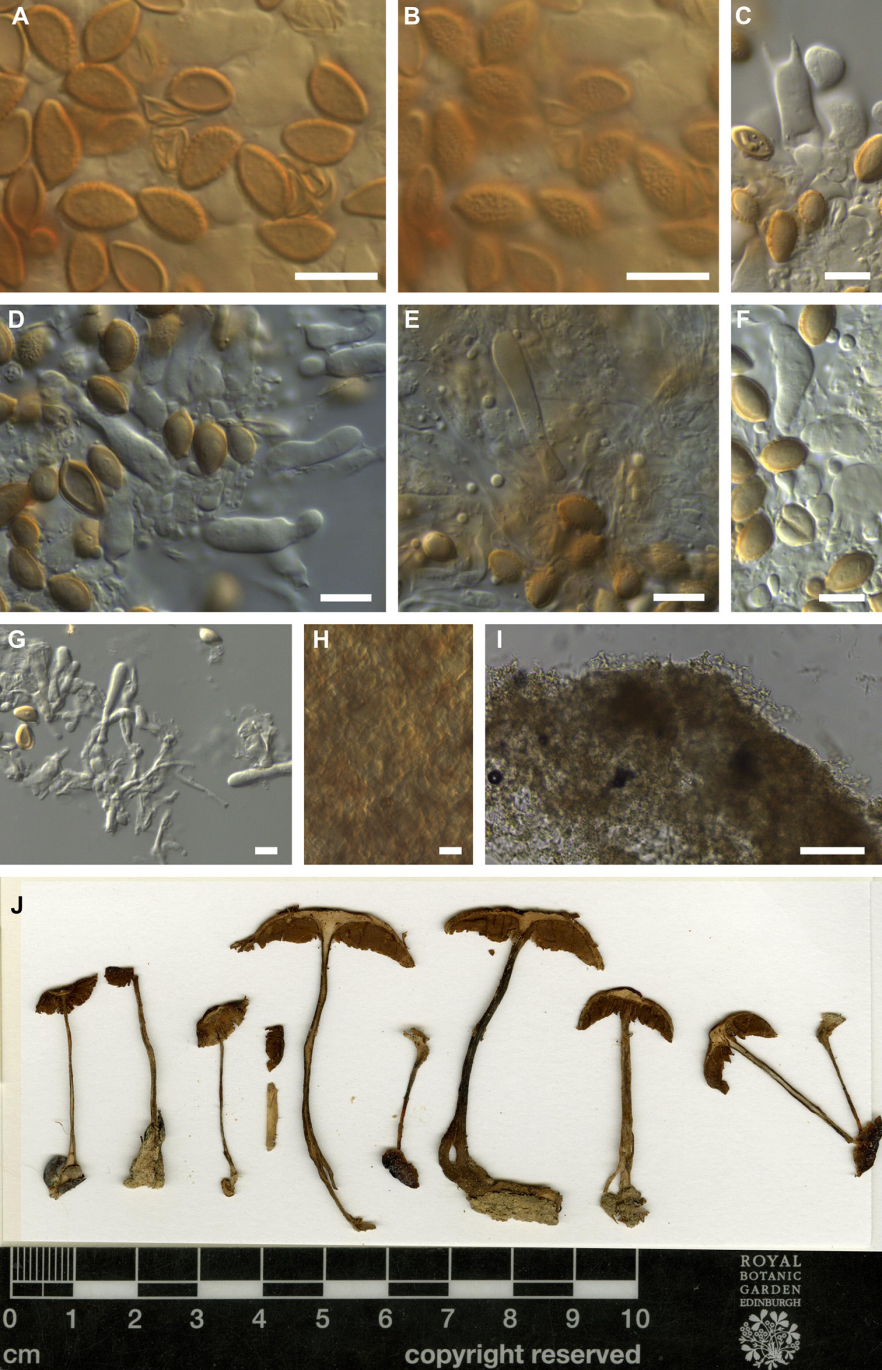
Microscopic features of *Hebeloma lactariolens* (E 00204780); intended holotype of *Psathyrella verrucispora* nom. inval.) **A** spores in Melzer’s reagent ×1600 **B** spore ornamentation in Melzer’s reagent ×1600 **C** basidium in KOH ×1000 **D** cheilocystidia in KOH ×1000 **E, F** pleurocystidium in KOH ×1000 **G** caulocystidia in KOH ×500 **H** sectional view of cutis below the gelatinous epicutis in KOH ×500 **I** sectional view of ixocutis showing thin gelatinous epicutis in KOH ×125. Scale bars: 10 µm (**A–H**), 100 µm (**I**). Photographs H.J. Beker. **J** Exsiccata (a section of photograph from http://data.rbge.org.uk/herb/E00204780 provided by the Royal Botanic Garden Edinburgh).

Clémençon and Hongo (1994) originally published this taxon as *Alnicola lactariolens* in the April issue of Mycoscience, apparently published on 1 Apr 1994; it appears Corner had effectively published the paper including the same taxon one day earlier, on 31 Mar 1994 as *Psathyrella verrucispora*. Both are morphologically clearly members of Hebeloma section Porphyrospora. The authors of both papers comment on the purple-brown (vinaceous) spore print, Corner (1994 [“1993”], p. 345) notes that the spore deposit color is fuscous purple, which is why he described his species in *Psathyrella* rather than *Lacrymaria*. Clémençon and Hongo (1994) commented on the spore deposit being a dark purple-brown color, an unknown feature of *Alnicola*. In Yang et al. (2005)*Alnicola lactariolens* was recombined into *Anamika* and later by Rees et al. (2013) into *Hebeloma*. The spore deposit color and its typical color change upon storage is the most striking feature of members of H. sect. Porphyrospora (Eberhardt et al. 2020). Good descriptions and further illustrations of *H. lactariolens* can be found in Corner (1994 [“1993”]) and Clémençon and Hongo (1994). Figure 6, shows various macro and micro characters of Corner’s intended type of *Psathyrella verrucispora*.

This species is rather variable molecularly and in the ML reconstruction forms a clade together with *H. youngii*, an Australian species growing with *Eucalyptus* and *Corymbia*, to our knowledge only known from the type locality (Rees et al. 2013). Even though the monophyly of *H. lactariolens* in relation to *H. youngii* is not bootstrap-supported within this analysis (Fig. 1), although it is in the BI results (see TreeBase), the molecular distance, the occurrence on different continents, the different host associations, and morphologically, the cheilocystidia which for *H. youngii* are more consistently lanceolate and the number of full length lamellae which for *H. youngii* is in the range 50–60 while for *H. lactariolens* is always less than 40, clearly separate these taxa. The Malaysian and Singapore records are from lowland tropical forests while the type has been described from a subtropical habitat from Japan, thus hinting at a wide climatic and geographical range. *Hebeloma lactariolens* is according to observations of S. S. L. not uncommon in Malaysia. The FRIM database includes additional records of this species (not studied) from Hutan Simpan Semangkuk, Fraser’s Hill, Pahang and the Pasoh Forest Reserve, Negeri Sembilan, from hill respective lowland dipterocarp forests.

### Hebeloma parvisporum

Taxon classificationFungiAgaricalesHymenogastraceae

Sparre Pedersen, Læssøe, Beker & U. Eberh., Mycologia 112: 179 (2020)

3FDD4FFC-1DEF-51C1-875B-8C00369F76F2

Figures 2C , 7

#### Type.

Laos. Xieng Khouang: Phoukhout, Laethong, ca. 1135 m a.s.l., 19.742408°N, 103.258102°E, on soil under Fagaceae, 18 Aug 2015, T. Læssøe, O.S. Pedersen (holotype: HNL [HNL 500968]; isotype: C! [C-F-122153]; database reference HJB14852; ITS GenBank Acc. No.: MK962004).

#### Heterotypic synonyms.

*Psathyrella splendens* Corner, Gdns’ Bull., Singapore 45(2): 341 (1994) [“1993”], nom. inval., Art. 40.7 ≡ *Lacrymaria splendens* (Corner) Voto, Boll. Assoc. micol. ecol. Romana 107: 95 (2019), nom. inval., Art. 40.7. Type. Singapore. Malay Peninsula, 9. Mar 1930, E.J.H. Corner (holotype: E! [E 00204835]; database reference HJB19597).

#### Other material examined.

Laos. Xiang Khouang: Khoun, Thoum, ca.1130 m a.s.l., 19.314945°N, 103.409749°E, under Fagaceae, 20 Aug. 2015, T. Læssøe, O.S. Pedersen (HNL [HNL 501009]; database reference HJB14850); Xiang Khouang: Paek, Phonekham, ca.1125 a.s.l., 19.494286°N, 103.269110°E, under Fagaceae, 16 Aug. 2015, T. Læssøe, O.S. Pedersen (HNL [HNL 500914]; database reference HJB17004); Xieng Khouang, Phoukhout, Ban Bong, ca.1150 m a.s.l., 19.672180°N, 103.135841°S, under Fagaceae 15 Aug. 2015, T. Læssøe, O.S. Pedersen (HNL [HNL 500884]; database reference HJB17007); Xieng Khouang, Phoukhout, Sui, ca. 1150 m a.s.l., 19.530514°N, 102.8659°E, under Fagaceae, 19 Aug. 2015, T. Læssøe, O.S. Pedersen (HNL [HNL 500984]; database reference HJB17005). MALAYSIA. Johor State, Mersing district, Endau-Rompin Selai, Endau-Rompin (Johor) National Park, Camp Lubuk Tapah, ca. 130 m alt., 2.2976°N, 103.1351°E, with *Dipterocarpus*, 19 Mar 2009, E. Horak 12795 (collection E. Horak at ZT; database reference HJB13362).

#### Remarks.

**Figure 7.**
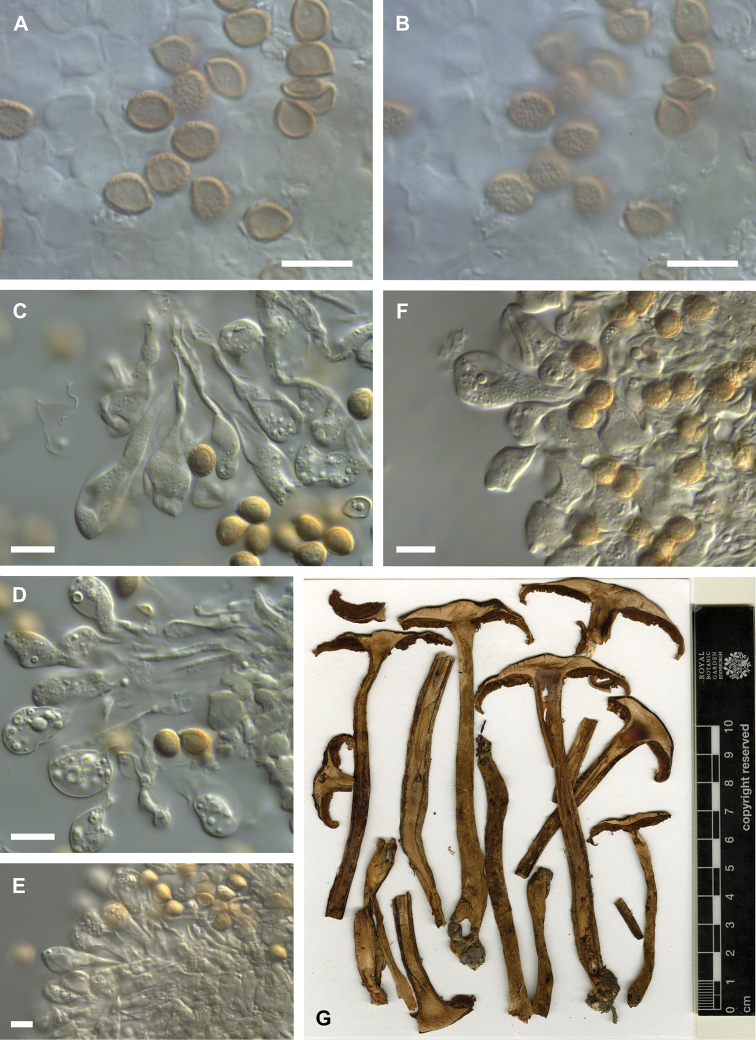
Microscopic features of *Hebeloma parvisporum* (E 00204835; intended holotype of *Psathyrella splendens* nom. inval.) **A** spores in Melzer’s reagent ×1600 **B** spore ornamentation in Melzer’s reagent ×1600 **C, D** cheilocystidia in KOH ×1000 **E** cheilocystidia and basidium in KOH ×500 **F** caulocystidia, in KOH ×1000. Scale bars: 10 µm. Photos H.J. Beker. **G** Exsiccata (a section of photograph from http://data.rbge.org.uk/herb/E00204835, provided by the Royal Botanic Garden Edinburgh).

The description of this species (Eberhardt et al. 2020) was based upon the above collections from Laos. The intended holotype of *P. splendens* was examined and is micro- and macromorphologically in agreement with *H. parvisporum*; this is illustrated in Fig. 7 which shows the main micro characters of Corner’s intended type. The collection from Malaysia is monophyletic with the Laos material. Molecularly, the species is most closely related to the Australian/New Zealand *H. victoriense* species group.

The collection cited as holotype for *P. splendens* was collected in Singapore while Corner also cites other collections from Singapore and Malaysia (Corner 1994 [“1993”]), to which we can add the Malaysian collection above. Plate 3 of Corner (1994 [“1993”]) illustrates the species macroscopically; Lee (2017) includes a photograph of *P. splendens* from the FRIM forest and comments that it often grows in large clusters and is common in the FRIM forest and other parts of the country. The FRIM database includes additional records of this species (not studied) from: Endau-Rompin National Park, Johor; Fraser’s Hill, Pahang; the FRIM grounds, Kepong, Selangor; Pasoh, Negeri Sembilan and Tasik Bera, Pahang from lowland and hill dipterocarp forests and a planted dipterocarp forest. S.S.L. observed this species also in degraded hill dipterocarp forest in Janda Baik, Pahang. The species is not listed on the checklist of mushrooms in Thailand (Chandrariskul et al. 2011), but Felix Hampe (oral communication, 21 Jan 2020) reported it from Thailand (Chiang Mai Prov.). Thus, it appears that this species may be widespread within tropical Asia, associated with Fagaceae and dipterocarps (*Dipterocarpus*). In Laos, *H. parvisporum* is found for sale in the local markets for human consumption, but its synonym *P. splendens* is not listed among the species consumed in Malaysia (Chang and Lee 2004; Samsudin and Abdullah 2019).

### Hebeloma radicans

Taxon classificationFungiAgaricalesHymenogastraceae

E. Horak, Beker & U. Eberh. sp. nov.

E0EC68CD-6813-5608-8200-137758EACEF6

838407

Figures 2D , 8 , 9

#### Diagnosis.

The combination of a deeply rooting stipe, about 60 full length lamellae (from stipe to margin of pileus) and spores where almost every spore has a strongly loosening perispore forming a clear layer around the spore, separate this taxon from all other members of H. sect. Porphyrospora, as does the ITS-sequence.

#### Type.

Malaysia. Johor State: Kluang district, Endau-Rompin Peta, Endau-Rompin (Johor) National Park, Kampung-Peta, trail to Kuala Marong, ca. 50 m a.s.l., 2.52°N, 103.36°E, on soil in lowland dipterocarp-oak forest, 3 Sept 2009, E. Horak, 13265 (holotype: collection E. Horak at ZT; isotype: FRIM [FRIM 62930]; database reference HJB13364, ITS GenBank Acc. No.: MT832018).

#### Description.

Basidiomes scattered. Pileus 37–64 mm wide, convex to broadly umbonate; surface dry or slightly viscid, without veil remnants on the pileus; cuticle color predominantly cream to pale buff (4A3, 4A4) in the center with paler margin, off-white to pale cream (4A2); pileus margin entire, hygrophanous. Lamellae adnate, moderately dense, thin, with approx. 60 full length lamellae and 2–3 lamellulae between the lamellae, off-white to cream when young, later pinkish or grayish red to purplish and eventually vinaceous to purple-brown following spore maturity; edges weakly fimbriate and white; the white edge remains when the basidiome is dried but the reddish brown color of the lamellae disappears with time. Stipe 160–194 mm long (including the ‘root’) and with central width 4–9 mm, cylindrical, distinctly and deeply rooting, white or alutaceous; surface dry, fibrillose, pruinose in the upper part, discoloring with handling and age. Flesh whitish, hardly discoloring where bruised. Smell fragrant; taste bitter. Spore deposit porphyry-brown (10E4). Exsiccata with no particular characteristics.

Basidiospores based on n = 94 spores of the holotype, 5% to 95% percentile range 8.7–10.2 × 5.6–6.6 µm, with median 9.5 × 6.2 µm and av. 9.5 × 6.2 µm with S. D. length 0.47 µm and width 0.34 µm; Q value 5% to 95% percentile range 1.43–1.65, with median 1.53 and av. 1.54 with S. D. 0.07; amygdaloid, with small apiculus and rounded apically, with a distinct thinning of the apical wall and never any sign of papilla, without guttules, usually very strongly ornamented, warty, with a strongly and distinctly loosening perispore on almost every mature spore (almost forming a uniform layer around the spore and making measurement quite difficult at times) and very strongly dextrinoid, immediately becoming deep and intensely red-brown in Melzer’s reagent, (O4; P3; D4); spore color under the light microscope distinctly brown. Basidia 21–29 × 6–8 µm, with av. 24.3 × 7.2 µm, cylindrical to clavate, without pigmentation, 4-spored. Cheilocystidia ventricose, primarily pyriform often mucronate or rostrate with width near apex (excluding any rostrum) 5% to 95% percentile range 5–8 µm, with median 6.4 µm and av. 6.5 µm with S.D. 1.06; and av. overall measurements 24 × 6.5 × 9.9 × 8.3 µm av. Cheilocystidium av. ratios A/M: 0.66, A/B: 0.79, B/M: 0.84. Pleurocystidia present, and abundant, and similar to cheilocystidia. Caulocystidia resembling the pleurocystidia but tending to be more cylindrical and longer. Pileipellis an ixocutis with a very thin epicutis only about 20 µm thick, with gelatinized hyphae up to 5 µm wide. The cutis below the epicutis is orange-brown and the trama below the cutis is made up of isodiametric cells up to 25 µm wide. Clamp connections at septa present throughout the basidiome.

#### Distribution.

Only known from the type locality in Endau-Rompin (Johor) National Park, Malaysia.

#### Ecology.

Scattered in lowland dipterocarp-oak woodland on the side of the path.

#### Etymology.

From ‘radicans’, meaning rooting, to emphasize this character of the species.

#### Remarks.

*Hebeloma radicans* with its vinaceous colored lamellae when mature and the porphyry colored spore print which turns brown with time, is a typical member of H. sect. Porphyrospora. The highly ornamented and highly dextrinoid spores are often seen in taxa of this section; while the consistently loosening perispore is also a common feature of a number of the taxa within this section, the regularity and presentation of the perispore is atypical and very distinctive. The rooting stipe is also unusual; while we have recorded rooting stipes in other members of this section, namely: *H. lactariolens*, *H. parvisporum*, and *H. victoriense*, in these cases it is a shallow root occurring infrequently and not on every basidiome. The rooting stipe of *H. radicans* is deep and more reminiscent of *H. radicosum*. This long rooting stipe should be sufficient to distinguish this species from other described members of this section, but taken together with the spore properties and also the moderately dense (but not crowded) lamellae (approx. 60 full length lamellae), assuming these characters are constant, this taxon is clearly distinct. In Fig. 1 as in the BI reconstruction, *H. radicans* is sister to the Oceanic *H. aminophilum* group clade, but this relationship is not supported. The ITS differs by at least 2.2% from other members of H. sect. Porphyrospora; there are many species in *Hebeloma* that are less distant from each other (Beker et al. 2016).

While, to date, we only have one collection of this species, given its morphological differences and molecular distinctness, we are confident that this taxon is different from any other described within *Hebeloma* and we hope that its publication will encourage its rediscovery. It is of course possible that it has been confused with other genera, e.g. *Psathyrella*, as was the case with other Malay Peninsula collections as described here, but thus far we have not been able to find any evidence of this.

**Figure 8.**
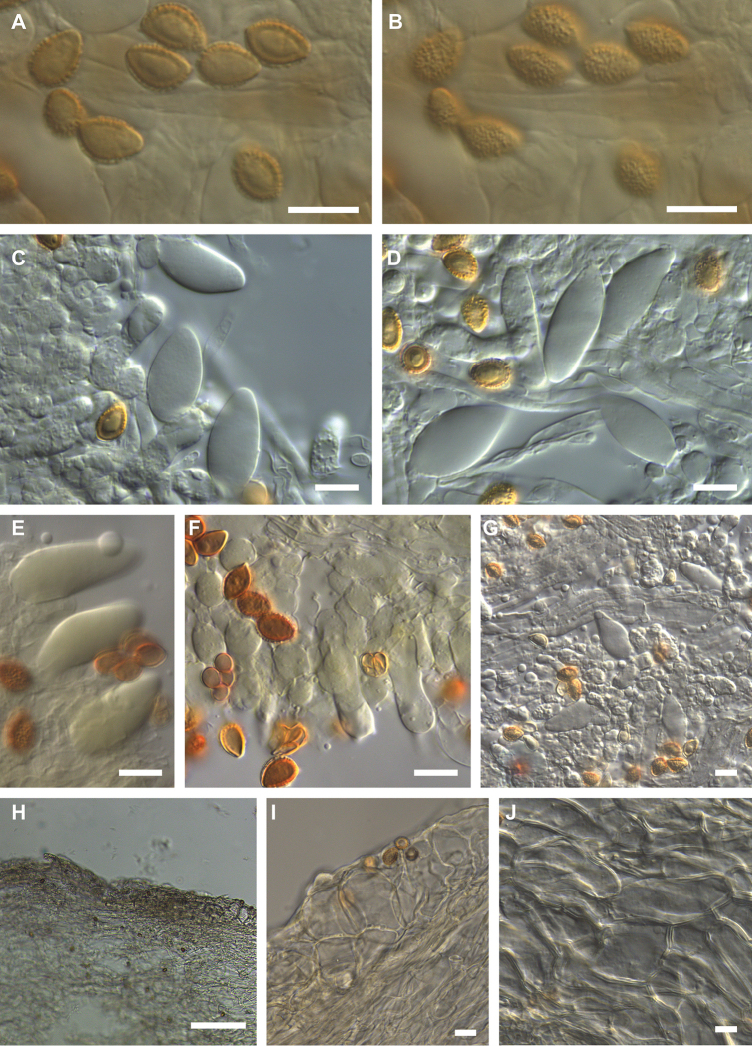
Microscopic features of *Hebeloma radicans* holotype (E. Horak 13265) **A** spores in KOH ×1600 **B** spore ornamentation in KOH ×1600 **C** cheilocystidia and basidium in KOH ×1000 **D** cheilocystidia and basidium in KOH ×1000 **E** pleurocystidia in Melzer’s reagent ×1000 **F** basidia in KOH ×1000 **G** pleurocystidia in KOH ×500 **H** sectional view of ixocutis showing thin gelatinous epicutis in KOH ×125 **I** sectional view of subcutis and trama below subcutis in KOH ×500 **J** sectional view of trama below subcutis in KOH ×500. Scale bars: 10 µm, 100 µm (**H**). Photographs H.J. Beker.

**Figure 9.**
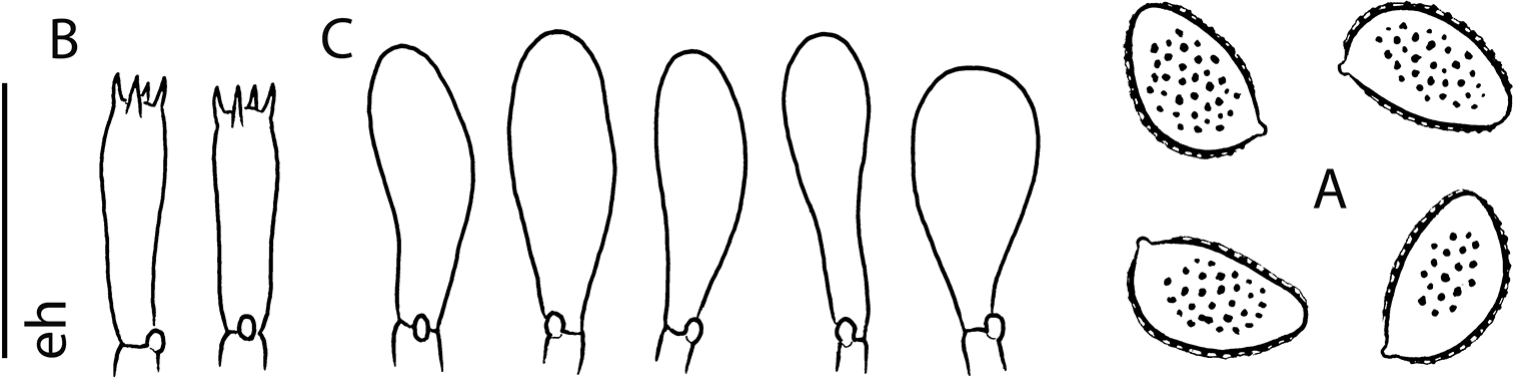
Microscopic features of *Hebeloma radicans* holotype (E. Horak 13265) **A** spores ×2000 **B** basidia ×1000 **C** cheilocystidia ×1000. Scale bar: 10 µm ×2000, 20 µm ×1000 and 40 µm ×500. Drawing E. Horak.

## Discussion

Had the describers of *Hebeloma parvisporum* been aware of *Psathyrella splendens*, they would have used that epithet for *H. parvisporum*. When describing the species, other genera like *Alnicola*, *Naucoria*, and even *Pholiota* were checked for misplaced *Hebeloma* species (Eberhardt et al. 2020), but it did not occur to the authors to investigate *Psathyrella* names – nor, it seems, to the authors who reclassified *Alnicola lactariolens* (Yang et al. 2005; Rees et al. 2013) without referring to *P. verrucispora*.

We here demonstrate the presence of four, presumably endogenous species, of *Hebeloma* in tropical forests of the Malay Peninsula, a genus previously overlooked in this region. In the checklist for Malaysia (Lee et al. 2012) the genus *Hebeloma* is missing. In fact, the entire group of ectomycorrhizal Hymenogastraceae is missing, unless one considers *Naucoria periniana*, adopted from Chipp’s checklist for the Malay Peninsula (Chipp 1921). This species was recombined into *Galerina* by Pegler (1975), thus outside of the ectomycorrhizal Hymenogastraceae, although it appears unlikely that Pegler and Chipp refer to the same taxon (Chipp, 1921 p. 383, “King’s collector”). *Hebeloma* is also missing from checklists for Singapore fungi (Tham and Watling 2017a–d). The ectomycorrhizal Hymenogastraceae are missing, if assuming that *Wakefieldia striaespora*, described from Singapore, does not represent the same genus as the Greek collections referred to as *Wakefieldia macrospora* (Kaounas et al. 2011), which are members of the Hymenogastraceae and have been sequenced from ectomycorrhizal root samples (Tedersoo and Smith 2013; Richard et al. 2011). *Hebeloma* and *Hymenogaster* records from Thailand (Chandrasrikul et al. 2011) appear to be from northern Thailand and are comprised of names of species that are presumably not native to Thailand (*H. albidulum*, *H. crustuliniforme*, *H. hiemale*, *H. radicosum*, *H. sacchariolens*, *H. sarcophyllum*); the single record of Hymenogaster cf. albellus (originally described from Tasmania by Massee 1898) would currently be referred to as *Descolea albella* and was moved to the Bolbitaceae (Kuhar et al. 2017). The cited collection of *H. angustilamellatum* from Thailand is not from the Malay Peninsula (the species is not listed by Chandrasrikul et al. 2011). Thus, it is a safe assumption that these are the first literature records of *Hebeloma* under this name from the Malay Peninsula, almost certainly endogenous species, and possibly also the first reliable records of ectomycorrhizal Hymenogastraceae. *Hebeloma* is considered rare in tropical forests. Apart from the records presented here, the only confirmed record is of *H. ifeleleretorum* (American Samoa, Kropp 2015).

Having said this, it should be noted that the authors of checklists for the Malay Peninsula (Lee et al. 2012; Tham and Watling 2017a–d) do state that these lists are not exhaustive, but represent the state of knowledge at the time of publication. Lack of opportunity and the generally overwhelming biodiversity has often prevented the investigation of less commercially important and generally less well-studied fungi. Those of us with field experience in the area have been aware of the presence of members of *Alnicola*, *Hebeloma* and *Hymenogaster* (probably also in the strict sense) on the Malay Peninsula for some time.

The molecular results support earlier results of Eberhardt et al. (2020) that the members of H. sect. Porphyrospora, originating from the western Pacific Rim, apart from *H. vinosophyllum*, form a well-supported clade. Within this clade, however, closely related species may be of Oceanic or southeast Asian origin, and may be associated with Fagaceae and/or dipterocarps or Myrtaceae. How this pattern came about, and even whether it will be supported when more data become available, is at this point an open question.

## Supplementary Material

XML Treatment for Hebeloma flavidifolium

XML Treatment for Hebeloma lactariolens

XML Treatment for Hebeloma parvisporum

XML Treatment for Hebeloma radicans
